# Effects of post exercise protein supplementation on markers of bone turnover in adolescent swimmers

**DOI:** 10.1186/s12970-020-00350-z

**Published:** 2020-04-15

**Authors:** Alexandros Theocharidis, Brandon J. McKinlay, Dimitris Vlachopoulos, Andrea R. Josse, Bareket Falk, Panagiota Klentrou

**Affiliations:** 1 Department of Kinesiology, Faculty of Applied Health Sciences, Brock University, 1812 Sir Isaac Brock Way, St. Catharines, Ontario L2S 3A1 Canada; 2 Children’s Health and Exercise Research Centre, College of Life and Environmental Sciences, University of Exeter, Exeter, UK; 3 Faculty of Health, School of Kinesiology and Health Science, York University, Toronto, Ontario Canada; 4 Centre for Bone and Muscle Health, Faculty of Applied Health Sciences, Brock University, St. Catharines, Ontario Canada

**Keywords:** PINP, CTXI, Bone turnover, High intensity swimming

## Abstract

**Background:**

This study examined the effects of whey protein supplementation, compared with an isocaloric carbohydrate beverage and water, consumed immediately following an intense swimming trial on bone turnover in adolescent swimmers.

**Methods:**

Fifty-eight (31 female, 27 male) swimmers (14.1 ± 0.4 years) were stratified into three groups matched for age, sex and body mass. The protein and carbohydrate groups consumed two isocaloric post-exercise beverages each containing 0.3 g^.^kg^− 1^ of whey protein (with ~ 6 mg of calcium) or maltodextrin while the control group consumed water. Participants provided a morning, fasted, resting blood sample, then performed an intense swimming trial consisting of a maximal 200 m swim followed by a high intensity interval swimming protocol (5x100m, 5x50m and 5x25m; 1:1 work-to-rest ratio). Following swimming, they consumed their first respective post-exercise beverage, and 2 h later, they performed a second maximal swim immediately followed by the second beverage. Approximately 3 h after the second beverage, two post-consumption blood samples were collected at 8 h and 24 h from baseline. Procollagen type 1 intact N-terminal propeptide (PINP) and carboxy-terminal collagen crosslinks (CTXI) were measured in serum. The multiples of medians of PINP and CTXI were also used to calculate bone turnover rate and balance.

**Results:**

No significant changes were observed in PINP. CTXI increased (+ 11%) at 8 h in all groups, but then significantly decreased (− 22%) at 24 h in the protein group only. The protein group also had a significantly higher calculated rate of bone turnover at 8 h and 24 h compared to baseline, which was not observed in the other groups.

**Conclusions:**

These results shed light on the potential importance of protein consumed shortly after intense swimming in promoting positive bone turnover responses up to 24 h following exercise in adolescent athletes.

**Clinical trial registration:**

ClinicalTrials.gov PRS; NCT04114045. Registered 1 October 2019 - Retrospectively registered.

## Introduction

Adolescence is characterized by acute bouts of accelerated growth of bone and muscle mass due to growth spurts [1]. Consequently, it is suggested that protein intakes for children and adolescents should be greater than adults’, relative to body size [2]. Specifically, from the USA and Canadian Dietary Reference Intakes, the recommended dietary allowance (RDA) for children 9–13 years of age is 0.95 g^.^kg^− 1^ per day, for adolescents 14–18 years of age is 0.85 g^.^kg^− 1^ per day and for adults over 19 years of age is 0.80 g^.^kg^− 1^ per day [3]. Although the beneficial effect of protein on bone, with or without exercise, has been previously documented in adults [4], no studies exist on the short or long-term effects of protein supplementation on bone turnover and bone development in children and adolescents. The potential effects of protein supplementation may be specifically important in athletes, as they generally have higher dietary needs. Only one study has previously reported a positive correlation between protein intake and bone growth [5] while protein deficiency during childhood and adolescence has been connected to suboptimal growth in height, weight, bone mass and overall protein in the body [5, 6]. Thus, the current protein RDA for children [3] are mostly derived from adult studies, trying to account for the needs of growth [7]. Importantly, since the RDA for protein refers to the average child, it may be insufficient to cover the energy and protein requirements of child athletes, who complete significant amounts of training and competition. In adults, protein needs of athletes have been reported to be higher than those of non-athletes [8, 9]. However, the protein requirements for child athletes or highly active children are still unspecified due the lack of studies examining the effects of protein consumption on bone accrual and bone turnover in young athletes, who may have higher needs for protein than non-athletic youth. This may be crucial for young athletes in low-impact sports like swimming who are perceived at an increased risk for suboptimal peak bone mass development because swimming is not an activity typically associated with bone-related benefits [10]. On the other hand, we recently demonstrated that in adults, low-impact exercise of higher intensity can lead to similar bone metabolism responses as high-impact exercise [11, 12], but there is no study of such comparison in children and adolescents. Furthermore, a systematic review of studies examining the effect of swimming on bone reported contradicting results, but overall, it seems that swimming does not stimulate bone growth above the habitual physical activity levels in children. In fact, a deleterious effect of swimming on bone mass has been suggested in some studies, due to the number of hours spent training in a low-impact activity [10]. A potential inadequate protein intake may exacerbate this deleterious effect on bone. The above evidence highlights the need for studies in this particular population with a focus on optimizing bone metabolism via supplementation.

Among adults, whey protein supplementation is often recommended following exercise for muscle growth due to its relatively fast absorption rate, which results in a more rapid stimulation in protein synthesis and recovery and a unique amino acid profile – i.e. higher leucine content per serving [13]. The appropriate timing of protein consumption, before or after exercise, has also been examined and showed that protein ingestion immediately after exercise provides a distinct advantage in stimulating muscle protein synthesis rate in adult populations [14, 15]. While protein supplementation has generally been examined in relation to muscle development and performance, protein may also play an instrumental role in other tissues which undergo stress during exercise. For example, according to a recent study in adult male endurance athletes, a protein beverage combined with carbohydrates consumed immediately post-exercise resulted in increased circulating levels of procollagen type 1 intact N-terminal propeptide (PINP), a bone formation marker, and decreased levels of carboxy-terminal collagen crosslinks (CTXI), a bone resorption marker [16]. One other study found that sole carbohydrate consumption provided after strenuous exercise also led to lower PINP and CTXI concentrations 2 h post-exercise, which then returned to baseline in the following days [17]. However, the effects of whey protein supplementation alone on bone turnover has not been examined, in either adult or youth athletes. Thus, considering that adolescence is the most important period for the attainment of peak bone mass and that relative energy requirements of youth athletes, compared to adults, are higher during and following exercise, it is possible that post-exercise whey protein supplementation may be beneficial to bone turnover.

This study examined whether whey protein, consumed immediately following an intense swimming trial, affects bone turnover markers in adolescent swimmers compared to both an isocaloric carbohydrate beverage and water. Specifically, we provided two post-exercise doses of 0.3 g^.^kg^− 1^ of whey protein each to adolescents, and examined changes in the serum concentrations of PINP and CTXI that are specific markers of type I collagen formation and degradation, respectively [18]. The dosage provided matches the relative intake (per kg body mass) previously used in adults post-exercise, although in absolute terms this is less than what has been used in adults [19]. It was hypothesized that lower-impact high intensity swimming, in combination with post-exercise protein supplementation would result in a) an increase in the circulating levels of both PINP and CTXI in the hours following the supplement consumption.

## Methods

### Participants

This study included data from 58 competitive swimmers (31 females and 27 males), 11-17 years of age, who trained and competed for at least 1 year, were free of injuries and any medical condition that prevented them from participating and were not taking medications or nutritional supplements. Swimmers were recruited from competitive swimming clubs across Southern Ontario as part of a larger study designed to examine the effect of post-exercise whey protein consumption on subsequent exercise performance, muscle damage, and inflammation. All study procedures were approved by our institutions’ Research Ethics Boards and Health Canada. Data and measurements presented in this study solely focus on bone turnover and have not been previously published.

### Study protocol

**Fig. 1:**
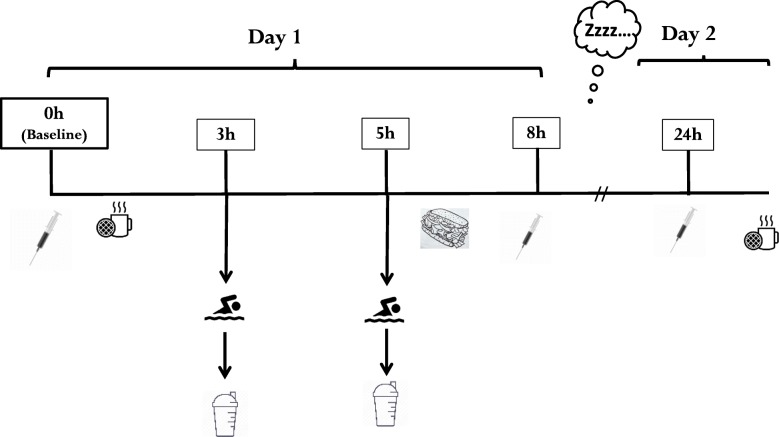
Study design

This was a double-blind, placebo control study, with participants stratified into three groups matched for age, body mass and sex as descripted below. An independent member of the research team was solely responsible for the group stratification and beverage preparation. The beverages were placed into opaque shaker bottles so both researchers directly involved with participants and the participants themselves were unaware of the beverage’s true contents. Participants were invited to the pool and laboratory for three visits as presented in Fig. 1. During the first visit (familiarization session), participants and their parents/guardians received detailed explanation on the purpose, duration, and goal of this study. They were informed of all tests and procedures and signed an informed consent/assent form. Subsequently, participants completed a screening questionnaire to report any injuries, allergies, and or health related conditions and anthropometric measurements were performed.

The second visit (testing day 1) included an intense swimming trial and an 8 h follow-up. This swimming trial was designed to simulate a competition session of multiple intense swimming bouts. Upon arrival in early morning (~ 0600 h), participants provided a fasted, resting venous blood sample. Following the baseline blood sample, they received a light, standardized breakfast of 300–400 kcal (depending on body mass). Approximately 45-60 min following breakfast, participants performed a warm-up of 1000 m low intensity swimming and a maximal 200 m freestyle-swim, followed by a high-intensity interval swim (HIIS) protocol, described below.

Within 0.5 h after the HIIS protocol (~ 3 h from baseline), the first post-exercise beverage was provided of either whey protein, an isocaloric carbohydrate beverage, or flavoured water, depending on their group assignment. Approximately 2 h after the first beverage (~ 5 h from baseline), participants performed a second 200 m freestyle maximum swim followed by a second beverage. Participants were then asked to complete several questionnaires, and for the remaining time participants attended lectures delivered by study personnel that related to the sport of swimming and the importance of nutrition in sports. A standardized lunch (a 12″ vegetable submarine sandwich) was also provided, which contained little protein, to minimize and control for additional protein intake before the follow-up blood draws. After lunch (i.e. ~ 3 h after the second beverage consumption), and 8 h from baseline, the first post-beverage blood sample was provided. The third visit was scheduled for the next morning (testing day 2), i.e., 24 h from baseline to provide a fasted, follow-up venous blood sample (Fig. 1). Participants were asked to refrain from training between visits and to self-record the food that they consumed between the two testing days.

Of note, the groups were matched for age and a similar split of male and female swimmers, with the latter being both pre-menarcheal and post-menarcheal. Given the potential for estrogen concentrations to affect bone metabolism, circulating estradiol concentrations were measured prior to the swimming trial.

### Intense swimming trial

All trials were performed in a 25 m pool. Following the 1000 m warm-up, swimmers performed a maximal 200 m front crawl swim. This test was performed in groups to simulate a swim competition trial. Following the maximal swim, swimmers performed the HIIS protocol, consisting of 5 × 100 m, 5 × 50 m, and 5 × 25 m freestyle sprints at near maximal effort, as determined by their split times (100 m) during the 200 m swim performance test. The interval swim protocol involved a swim to rest ratio of 1:1.

### Protein consumption

The protein group (*n* = 21) was provided with two servings (beverages) of chocolate-flavoured whey protein; the carbohydrate group (*n* = 19) was provided with two isocaloric chocolate-flavoured Maltodextrin servings; and the placebo control group (*n* = 18) was provided with chocolate-flavoured water containing no sugar. The protein, carbohydrate, and placebo beverages were of similar volume and were served in opaque shaker cups, prepared and pre-packaged by an independent assistant, so that both the researchers and the swimmers were blinded to the content of the beverages (double-blind).

The protein and carbohydrate beverages were prepared individually, such that each serving was 0.3 g (of whey protein or maltodextrin) per kg body mass. For example, a participant of 40 kg received 12 g of protein (isolate required, 15 g) or carbohydrate in each beverage. The protein dose was chosen based on a previous study using oral tracer methods [20], which found that post-exercise ingestion increases net whole-body protein balance in favour of synthesis in a dose-dependent manner with larger protein intakes (e.g ~ 0.32 g^.^kg^− 1^) required to sustain a net anabolic environment over a 24 h period. The whey protein used was BiPRO [21]. In terms of specific ingredients listed, each 25 g scoop of the isolate contains 0.5 g of fat, 170 mg of sodium, 130 mg of potassium, 1 g of carbohydrate, 1 g of fiber, 20 g of protein and 10 mg of calcium. Although calcium is known to have positive effects on bone turnover markers our participants received two beverages of ~ 15 g of whey protein each (i.e., ~ 6 mg of calcium per beverage) totalling ~ 12 mg of extra calcium for the day, which is a very small amount given the RDA of 1300 mg of calcium in this age group [3] and the average daily consumption > 1300 mg in all groups (Table 1).

**Table 1:** Age, maturity offset, physical characteristics, dietary intake and training characteristics of participants in each group. Data are presented as mean ± SEM

Variables	Protein (***n*** = 21)	Carbohydrate (***n*** = 19)	Water (***n*** = 18)	***P*** value
Number of Females / Males	11/10	11/8	9/9	
Age (years)	13.4 ± 0.3	14.3 ± 0.4	14.0 ± 0.4	0.17
Years from age of PHV (years)	−0.46 ± 0.3	0.43 ± 0.3	0.21 ± 0.4	0.15
Baseline Estrogen (pg^.^ml^− 1^) (females only)	10.7 ± 3.8	10.1 ± 2.3	8.6 ± 2.1	0.89
Height (cm)	160.4 ± 2.7	165. ±2.2	165.7 ± 2.3	0.24
Body Mass (kg)	51.6 ± 3.0	56.7 ± 2.3	55.2 ± 3.6	0.45
Relative Body Fat (%)	16.5 ± 1.4	16.5 ± 1.4	15.2 ± 1.7	0.79
Dietary Energy Intake (kcal^.^ kg^-1.^day^−1^)	43.3 ± 3.9	47.9 ± 8.0	48.2 ± 6.4	0.81
Dietary Protein Intake (g^.^kg^-1.^day^−1^)	1.7 ± 0.2	1.9 ± 0.3	1.9 ± 0.3	0.71
Dietary Calcium Intake (mg^.^kg^-1.^day^− 1^)	1351 ± 127.6	1648 ± 147.6	1518 ± 158.7	0.34
24 h Energy Intake^a^ (kcal^.^kg^-1.^day^− 1^)	55.2 ± 4.9	51.5 ± 4	53.3 ± 3.9	0.83
24 h Protein Intake^a^ (g^.^kg^-1.^day^− 1^)	1.4 ± 0.1	1.3 ± 0.1	1.4 ± 0.1	0.73
24 h Calcium Intake^a^ (mg^.^kg^-1.^day^− 1^)	1340 ± 80	1366 ± 81.1	1365 ± 127.6	0.98
Training Experience (years)	4.6 ± 1.4	5.2 ± 1.7	4.8 ± 1.9	0.68
Training Frequency (sessions·wk.^− 1^)	5.6 ± 1.3	6.4 ± 0.9	5.5 ± 1.4	0.09

PHV=Peak Height Velocity. The 24 h values refer to the nutritional intake during the study duration. ^a^The 24 h values refer to the intake during the study duration and both the protein and nutritional intake values do not include the supplements received

During the 24 h between testing days, participants were asked to self-record their food consumption, in order to control for potential differences among groups in terms of dietary energy and protein intake above and beyond the beverages received. Indeed, there were no differences between groups in either the habitual dietary intakes or the intake during the 24 h duration of the study (Table 1). During the 24 h of the study all groups reported lower protein intake than what they reported to habitually consume prior to the study (1.3–1.4 versus 1.8–1.9 mg^.^kg^-1.^day^− 1^, respectfully). This difference may be due to the standardized non-protein breakfast and the non-protein lunch provided during the experimental day. Thus, the difference among the groups in terms of the protein intake was indeed the total 0.6 mg^.^kg^-1.^day^− 1^ of whey protein consumed by this group.

### Measurements

#### Baseline measurements

Body mass was measured using the InBody520 bioelectrical impedance analysis system (Biospace.228, Los Angeles, CA, USA) to the nearest 0.1 kg. Height and sitting height were measured using a Seca 213 Portable Stadiometer (CME Corp., Warwick, RI, USA) to the nearest 0.1 cm. Measurements were taken wearing light clothing and no shoes. Maturity offset (years from the age of peak height velocity) was calculated based on height, sitting height and body mass measurements, as described by Mirwald et al. [22].

In addition, study participants had to describe the level of competition, the number of years participating in that sport as well as exercise sessions per week and workout duration. To evaluate dietary intake, the swimmers filled out the Block Questionnaire that is designed to assess dietary habits through a recall of foods eaten in the last 6 months [23]. We specifically used the adult version of the Block Questionnaire, which we have previously used in adolescent populations [24]. Parental assistance was allowed, if needed. All responses were analyzed by NutritionQuest (Berkley, CA., USA). Food consumption across the 24 h testing period was self-reported using a 24 h food-record, which was then analyzed using ESHA nutritional software [25].

#### Blood analysis

Blood samples were aliquoted and stored in a -80 °C freezer until blood analysis. Serum and plasma were stored in labelled 1.5 mL Eppendorf tubes. After every blood collection, hematocrit was measured [26]. This measurement is important to determine exercise-induced changes in plasma volume, which can affect serum concentrations [27]. Relative change in plasma volume (%ΔPV) was estimated using the Van Beaumont equation below [28]:

$$ \%\varDelta PV=\frac{100}{100- Hct1}+\frac{100\left( Hct1- Hct2\right)}{Hct2}\% $$

Where Hct1 is hematocrit at baseline, and Hct2 is hematocrit at each post baseline measurements. The %ΔPV was used to adjust serum concentrations of CTXI and PINP after the multiple bouts of exercise 8 h and 24 h from baseline.

The bone markers measured are both matrix-derived markers recognized by both the International Federation of Clinical Chemistry and Laboratory Medicine (IFCC) and the International Osteoporosis Foundation (IOF) as the main markers to predict future osteoporosis [29]. PINP is a well-accepted marker of bone formation, produced by the formation of type I procollagen by osteoblasts [30] and CTXI is one of the main bone resorption markers released from the breakdown of type I collagen in the initiation of the bone turnover process [31]. Serum concentrations of CTXI and PINP were measured in duplicate by standard laboratory techniques on a microplate reader at 450 nm optical density. CTXI concentrations were measured by an immunochemiluminometric assay (Elabscience, China). The CTXI assay detection range was 0.16–10 ng/ml. The CTXI inter-assay coefficient of variation (CV) was 9.33% and the intra-assay CV was 11.62%. Serum PINP was assessed using a radioimmunoassay (Bioassay Technology laboratory, Shanghai, China). The PINP inter-assay CV was 7.58%, intra-assay CV was 3.85%. Serum PINP concentrations were measured in ng/ml. The normal detection range for this assay was 5–2000 ng/ml. Serum concentrations of estradiol were also measured in duplicate using an ELISA assay (human estradiol E2 kit, Abcam, Toronto, Ontario, Canada). The estradiol inter-assay CV was 5.24%, intra-assay CV was 8%. a sensitivity of 10–1000 pg/ml with 101.3% recovery in serum.

In addition, we applied the method by Bieglmayer & Kudlacek [32] to calculate the balance and the rate of bone turnover as indirect indices of the overall effect on bone. This method compares the multiple of medians (MoM) of a formation (PINP) and a resorption (CTXI) marker, which provide a measure of how far an individual’s result deviates from the median. Briefly, the MoM was calculated for each marker using the serum concentrations within each group at the respective timepoint. The bone turnover balance at a specific time point was then calculated as: Bone Turnover Balance = MoM_F_/MoM_R_, where MoM_F_ is the MoM of PINP as the formation marker and MoM_R_ is the MoM of CTXI as the resorption marker [32]. The MoM values were also used to calculate bone turnover rate, i.e., how fast or slow the turnover occurs, based on the following equation: Bone Turnover Rate = √(MoM_F_^2^ + MoM_R_^2^) [32]. This standardization method represents the balance of bone formation and resorption, together with the rate of bone turnover, although it does not necessarily reflect the bone remodeling unit and is not a direct assessment at the tissue level. The utility of this standardization method to indicate the overall balance between bone resorption and formation has been previously shown in adults [33–36], although there is no evidence about their use in children and adolescents.

### Statistical analysis

Statistical analyses were performed using SPSS version 25.0 for Windows. There were 4 missing samples from a total of 174 blood samples used in this study (58 participants × 3 sampling times). Missing values (4 out of 174 cases for PINP and 4 out of 174 cases for CTXI) were replaced with the group mean value at the corresponding time point [37]. Outlier values, i.e., z-scores above or below two standard deviations (13 out of 174 cases for CTXI and 8 out of 174 cases for PINP), were trimmed with less extreme values using the group mean value ±2 standard deviations at the corresponding time point. This outlier treatment has been previously recommended to retain their extremeness within the range limits of the dataset and help to normalize the data [38, 39]. Data were then screened for normality using the Kolmogorov-Smirnov test, z-scores for skewness and kurtosis of ±3 and visual screening of histograms for symmetry. The screening showed that CTXI and PINP, as well as the bone turnover rate and balance, were not normally distributed and were therefore log-transformed for the analysis. Figures present absolute values, not log-transformed.

A series of one-way ANOVAs were performed to determine whether there were any group differences in anthropometric measures, training, as well as habitual energy and protein intake at baseline A three-way (time-by-group-by-sex) analysis of variance for repeated measures (RM-ANOVA) was performed to assess changes in the CTXI and PINP concentrations after both beverages were consumed, i.e., at 8 h and 24 h, relative to their concentrations at rest. Specifically, in order to overcome the non-uniformity bias when comparing individuals who differ in absolute concentrations, the CTXI and PINP concentrations at 8 and 24 h were expressed as a percentage of their concentration at baseline. In the event of a significant main effect or interaction, pairwise *post-hoc* comparisons with appropriate Bonferroni adjustment were performed. An alpha value of *p* < 0.05 was used to determine statistical significance.

## Results

There were no significant differences between groups in terms of age, maturity offset, resting estrogen concentrations for females, physical characteristics, training volume and dietary intake (Table 1). Table 2 presents the absolute concentrations of CTXI and PINP for each group and at each time point. Although non statistically significant, there was a 16–42% difference in the baseline concentrations of PINP and 18–20% difference in the baseline concentrations of CTXI amongst the groups. Therefore, the CTXI and PINP concentrations at 8 h and 24 h were expressed relative to their baseline for further analysis.

**Table 2:** Circulating levels of bone turnover markers and indices of bone turnover rate and balance for each group (protein, carbohydrate, water) at baseline, i.e. morning, fasted resting concentrations, as well as at 8 and 24 h. Data are presented as mean ± SEM

Variables	Protein (***n*** = 21)	Carbohydrate (***n*** = 19)	Water (***n*** = 18)	Between Groups (***P*** value^**!**^)
**CTXI (ng**^**.**^**ml**^**−1**^**)**				
Baseline	0.46 ± 0.07	0.56 ± 0.09	0.45 ± 0.08	0.58
8 h	0.54 ± 0.07	0.56 ± 0.08	0.42 ± 0.06	0.44
24 h	0.42 ± 0.07	0.55 ± 0.09	0.41 ± 0.07	0.42
**PINP (ng**^**.**^**ml**^**−1**^**)**				
Baseline	376.7 ± 108.1	650.0 ± 158.1	547.5 ± 131.5	0.32
8 h	403.5 ± 122.1	678.1 ± 153.2	547.0 ± 133.1	0.32
24 h	362.1 ± 104.8	581.7 ± 129.9	535.4 ± 132.5	0.35
**BT Balance**				
Baseline	2.0 ± 0.6	2.2 ± 0.7	2.7 ± 0.9	0.84
8 h	2.8 ± 0.9	2.1 ± 0.6	1.7 ± 0.6	0.64
24 h	2.4 ± 0.8	2.0 ± 0.7	2.2 ± 0.8	0.95
**BT Rate**				
Baseline	2.9 ± 0.6	2.4 ± 0.3	2.4 ± 0.3	0.76
8 h	3.8 ± 0.9	2.3 ± 0.3	1.9 ± 0.2	0.09
24 h	2.4 ± 0.7	2.0 ± 0.3	2.2 ± 0.3	0.16

*CTXI* Carboxy-terminal collagen crosslinks, *PINP* Procollagen type 1 intact N-terminal propeptide, *BT Balance* Bone turnover balance calculated as MoMF/MoMR, where MoM is the Multiple of Median of formation (MoM_F_) and resorption (MoM_R_), *BT Rate* Bone turnover rate calculated as √(MoM_F_^2^ + MoM_R_^2^). ^!^One-way ANOVAs were performed using log transformed values

**Fig. 2:**
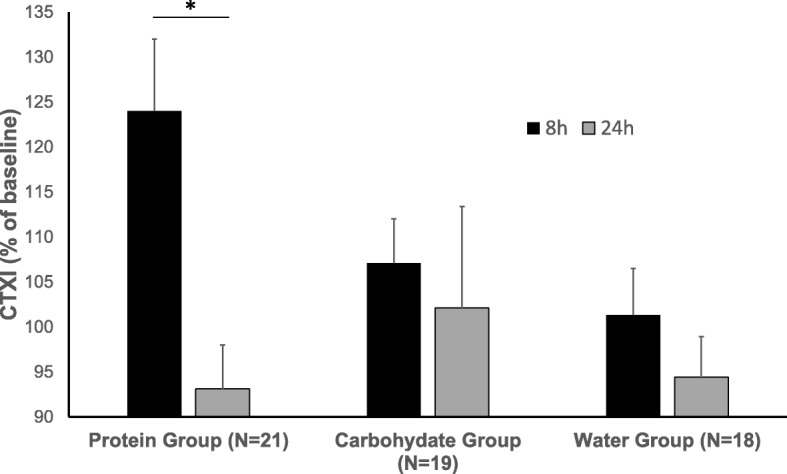
Relative changes in serum concentrations (mean ± SEM) of carboxy-terminal collagen crosslinks (CTXI) following the two post-exercise supplement beverages (i.e., 8 h and 24 h from baseline) in adolescent female and male swimmers, where baseline values are taken as 100%. There was a significant main effect for time (F = 11.48, *p* = 0.001, η_p_^2^ = 0.18) and a significant time-by-group interaction (F = 4.88, *p* = 0.001, η_p_^2^ = 0.16), reflecting a significant decrease (*p = 0.04) from 8 h to 24 h in the protein group only

**Fig. 3:**
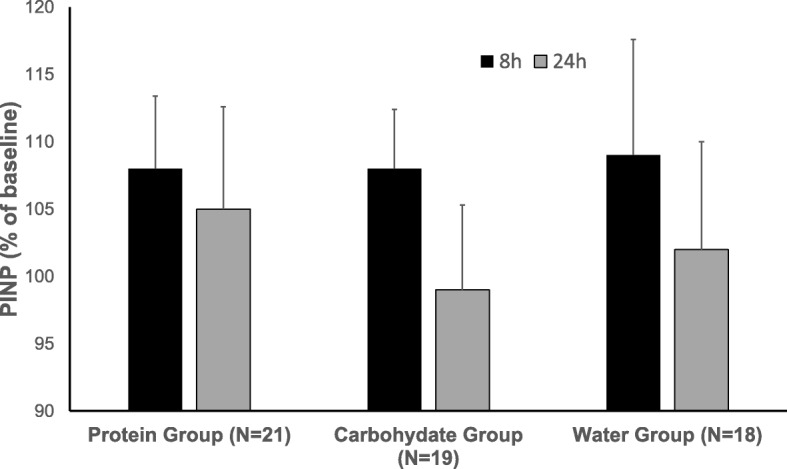
Relative serum concentrations (mean ± SEM) of procollagen type 1 intact N-terminal propeptide (PINP) following the two post-exercise supplement beverages (i.e., 8 h and 24 h from baseline) in adolescent female and male swimmers, where baseline values are taken as 100%. No significant main effects were observed for time, group or sex, and no significant interactions

For the relative CTXI concentrations at 8 and 24 h, a significant main effect for time was observed, reflecting an overall 11% increase at 8 h. Furthermore, we also found a significant time-by-group interaction reflecting a 22% significant decrease at 24 h in the protein group only (Fig. 2), with no other significant post-hoc differences between groups. No significant main effects or interactions were observed for PINP across time or between groups and sexes (Fig. 3).

The bone turnover balance scores derived from Bieglmayer & Kudlacek [32] confirmed that the balance was > 1 (i.e., formation>resorption) at rest and consistently over time, with no differences between groups (Table 2). This is to be expected due to the age of the participants, as bone formation exceeds bone resorption during growth and development [37]. Furthermore, there was a significant time-by-group interaction found for the calculated bone turnover rate, reflecting significantly higher values compared to baseline at 8 h (+ 32%) and 24 h (+ 18%) in the protein group, with no differences over time in the carbohydrate group and significantly lower values at 8 h in the water group (Table 2).

## Discussion

This is the first study to examine the effects of whey protein versus carbohydrate consumption immediately following exercise on bone turnover markers (PINP, CTXI) in either athletic or non-athletic youth. The most important finding is the observation that 8 h from baseline, after the swimmers performed a high-intensity swim session and received the full supplement amount, there was an overall significant increase in CTXI, which then significantly dropped at 24 h only in the protein group. Although there are limitations about what these circulating markers of bone turnover can tell us about skeletal site-specific responses, i.e., they can only be suggestive, without reflecting the bone remodeling unit, the combined findings of an acute decrease in CTXI without changes in PINP following whey protein consumption indicates that this could be an effective strategy to stimulate bone turnover and increase bone accrual over time.

Our results of CTXI increasing early after post-exercise protein supplementation followed by its significant decrease at 24 h only in the protein group is a novel finding. An increase in CTXI has been previously shown in adults immediately post-exercise, in absence of supplementation [12]. Thus, our CTXI findings agree with previous studies in adults and suggest that, even with supplementation, CTXI increases may remain for at least 2 h. Previously, protein combined with carbohydrates consumed immediately after exercise decreased CTXI immediately after an exhaustive run in endurance-trained male adults [16]. The difference in findings between the two studies may be because of the different impact of the exercise (low- versus high-impact), or the different age of the athletes, which could also mean a different training experience and volume. Specifically, the adult participants were trained endurance runners who had been training consistently for a minimum of 2 years and for an average running distance of 49.9 ± 12.5 km/week [12], while our participants had been training for an average of 5 years and 6 sessions per week (10–12 h/week). It is possible that during growth, responses to exercise and dietary supplements differ between child and adult populations.

PINP did not show any significant differences across time in any of the three groups. This result is supported by previous literature documenting that formation markers are less responsive to acute bouts of exercise than resorption markers [40, 41]. It is possible that 24 h may not be sufficiently long to see a significant change in PINP. On the other hand, the absence of a PINP response is in line with the notion that low-impact activities like swimming do not provide sufficient mechanical loading to stimulate bone formation in this age group [10], and this seems to be true irrespective of its high intensity and the supplementation. A previous study in adults reported an immediate increase in PINP following intense running with post-exercise consumption of a protein combined with a carbohydrate beverage [16]. Specifically, this study reported PINP concentrations to significantly increase by the end of intense treadmill running (at 75% of VO_2_max to exhaustion), but then decreasing to below baseline levels by 1 h post-exercise in all trials, irrespective of the type of beverage. However, in addition to the high-impact activity, the post-exercise energy intake was higher in this adult study. Additionally, we did not have a swimmer group who consumed a combined beverage. Our groups consumed either whey protein or carbohydrate and did not show any significant change in PINP post-exercise. It is possible that there is an additive effect of the protein and carbohydrate combined consumption, along with the higher total energy intake. An alternative reason for not finding acute time and group effects on the PINP response might be that exercise has cumulative effects on bone formation and that these effects may be apparent only following longer-term training and post-exercise protein intake. For example, a recent study in young men has shown increases in PINP after 12 weeks of resistance training and post-exercise supplementation of high-protein yogurt but no change in PINP following just 1 week of training [42]. Additionally, it should be noted that our participants were adolescents, in whom formation markers of bone turnover are potentially already chronically elevated, reflecting the high rate of linear growth and bone accretion [43]. Specifically, PINP has been reported to reach its highest concertation in 14 years old boys [44]. Thus, it seems logical that the benefits of protein supplementation on bone turnover are more apparent in the process less activated during growth, i.e., bone resorption.

Sale et al. (2015) [17] found that, in young adults, carbohydrate consumption provided after strenuous exercise led to lower PINP and CTXI concentrations 2 h post-exercise, which then returned to baseline in the subsequent days. In the present study, PINP and CTXI levels were used to calculate balance and rate, as described by Bieglmayer and Kudlacek [32]. In addition to the delayed drop in CTXI, the protein group also had a significantly higher calculated rate of bone turnover at 8 h and up to 24 h post baseline, which was not observed in the other groups. The carbohydrate group, which consumed an isocaloric beverage, seems to have maintained bone turnover rate, while the control group, which consumed only flavoured water (non-caloric beverage), showed a small but significant decrease in bone turnover rate from baseline to 8 h and up to 24 h. Since the groups had no differences in energy and macronutrients consumed during the study, we can speculate that the difference between the control and carbohydrate group is in the extra energy consumed from the beverage (i.e. ~ 144 kcals), which appears to have been sufficient to maintain the levels of bone turnover, while the whey protein, along with the added calcium (~ 6 mg per beverage), may have had an additional beneficial effect on bone at the 24 h time point. Interestingly, during the 24 h of the study all groups reported lower protein intake than what they reported to regularly consume prior to the study. This difference could be a factor in the results, especially for the water group, and can probably be attributed to the standardized non-protein breakfast and the non-protein lunch provided during the course of the study.

Our findings on the beneficial effects of whey protein are supported by results of previous studies showing that protein promotes bone deposition in children, increases bone turnover in adults and minimizes bone loss in elderly people [45–47]. This increase in bone turnover is important in younger populations as it is believed that higher rates of bone turnover are associated with greater bone growth [43]. However, there are no previous studies using protein supplementation combined with exercise to examine bone turnover markers in children. There is only one previous study that examined the chronic response of 10 g versus 20 g of protein supplementation in New Guinea children with the control group following the typical (low-protein) diet served at school, which involves mostly vegetable sources of protein, and specifically, 50% of protein intake from sweet potatoes [45]. After 8 months, they found that the children who received 20 g of protein showed greater increments of growth (height and weight) and skeletal maturation compared to the control group consuming the traditional low protein diet. Although this study is not a direct comparison to ours, since it examined chronic supplementation and it did not involve exercise, the results of both studies combined suggest that protein supplementation has the potential to positively affect bone in children.

A major strength of this study lies with the specific population assessed, as swimmers are suspect for being at risk for suboptimal bone accretion, due to the low-impact nature of the activity [10]. Adolescence is a crucial period for bone mineral accrual [1], so studying the combined effects of intense training and protein consumption on bone metabolism in a population that may need it more is important. Another strength of this study is that the three groups were matched for age, sex and body mass, all of which could potentially affect bone metabolism. In addition, all measurements were done in the morning and after a standardized breakfast, thus eliminating diurnal fluctuations and nutritional effects. This was important because CTXI is known to be at its peak during morning hours and influenced by food intake [31].

The study also has its limitations. An intrinsic limitation of this study is that CTXI and PINP are measured in serum, which limits their utility in providing a mechanistic understanding of any changes in the bone unit locally. Another potential limitation is that the whey protein isolate contained ~ 6 mg of calcium per beverage, which arguably may have contributed to the effect on bone at the 24 h time point. However, this is a very small amount given the high RDA for calcium in this age group (1300 mg), and since all groups were above the RDA during the 24 h of the study, it is unlikely that the effect of the additional 12 mg of total calcium received during the day was impactful. Of note, despite the observed positive post-exercise changes in the protein group marked by a decrease in CTXI from 8 h to 24 h, this was only an acute trial. It does not allow for drawing conclusions regarding potential long-term benefits of protein supplementation on bone. Future studies are needed to determine the effects of long-term protein supplementation and exercise on bone metabolism in young athletes. Another limitation of the study is that, while we used two beverages of 0.3 g^.^kg^− 1^ of body mass each, it is unknown whether this is the optimal dose of whey protein to achieve maximal effect on bone. Future research should examine the effects of different doses of protein on bone in youth.

## Conclusions

The consumption of two whey protein beverages following an intense swimming trial resulted in a significant delayed decrease in CTXI from 8 h to 24 h-post exercise, which followed its initial post-exercise increase, and no change in PINP for up to 24 h in adolescent swimmers. Any lifestyle strategy that can promote bone accretion in adolescent swimmers is beneficial because it can lead to a higher peak bone mass and improvement in bone mineral density in the long term. Future research should focus on the long-term benefits of protein consumption on bone turnover markers in combination with a chronic exercise training in children and adolescents, especially those who participate in demanding, low impact sports.

## Data Availability

The data that support the findings of this study are available on request from the corresponding author [PK]. The data are not publicly available due to REB restrictions.
